# Supplementary material for: Systems Biology of Recombinant 2G12 and 353/11 mAb Production in CHO-K1 Cell Lines at Phosphoproteome Level
Source: Proteomes. 2025 Feb 10;13(1):9. doi: 10.3390/proteomes13010009 (PMC11843875; doi:10.3390/proteomes13010009)
Supplement: Supplementary file 1 [file proteomes-13-00009-s001.zip › proteomes-3312466-Figure S1.pdf]

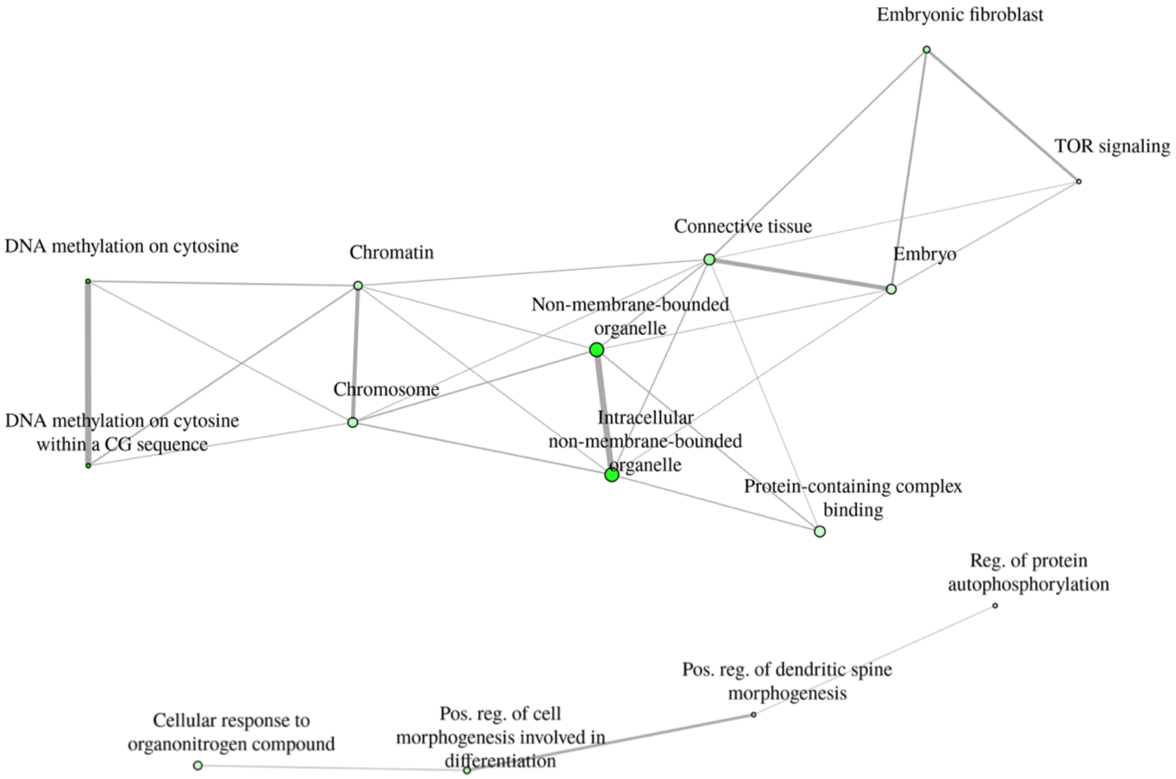

Figure S1. The enrichment plot network analysis comparing 353/11_TM and 353/11 illustrates the top 15 identified terms across different functional, using an edge cut-off of 0.3. Nodes correspond to the enriched terms, with edges representing their interactions. The thickness of the edges reflects the degree of gene overlap, while node size indicates the number of genes associated with each term (Table S10).
